# Minimally invasive right colectomy anastomosis study (MIRCAST): protocol for an observational cohort study of surgical complications using four surgical techniques for anastomosis in patients with a right colon tumor

**DOI:** 10.1186/s12893-020-00803-x

**Published:** 2020-07-13

**Authors:** Marcos Gomez Ruiz, Paolo Pietro Bianchi, Sanjay Chaudhri, Roger Gerjy, Ismail Gögenur, David Jayne, Jim S. Khan, Tero Rautio, Luis Sánchez-Guillén, Giuseppe Spinoglio, Alexis Ulrich, Philippe Rouanet

**Affiliations:** 1 Unidad de Cirugía Colorrectal, Servicio de Cirugía General y Aparato Digestivo, Hospital Universitario Marqués de Valdecilla, Av. Valdecilla s/n, 39008 Santander, Spain; 2 IDIVAL, Instituto de Investigación Sanitaria, 39008 Santander, Spain; 3 Department of Surgery, Division of General and Minimally-Invasive Surgery, International School of Robotic Surgery, Clinical Robotic Surgery Association (CRSA), Ospedale La Misericordia, Via Senese 170, 58100 Grosseto, Italy; 4 Leicester General Hospital, University Hospitals Leicester NHS Trust, Leicester, UK; 5 Department of Surgery, Danderyd University Hospital, 182 88 Stockholm, Sweden; 6 Department Surgery, Center for Surgical Science, Zealand University Hospital, Institute for Clinical Medicine, Copenhagen University, Copenhagen, Denmark; 7 Surgery, Level 7 Clinical Sciences Building St James’s University Hospital, Leeds, LS9 7TF UK; 8 Portsmouth Hospitals NHS Trust, University of Portsmouth, Portsmouth, UK; 9 Anglia Ruskin University, Chelmsford, England; 10 Department of Surgery, Oulu University Hospital, PL 21 OYS, 90029 Oulu, Finland; 11 Department of Surgery, General University Hospital Elche, University Miguel Hernández, Camí de l’Almazara 11, CP 03203 Elche, Spain; 12 Digestive Surgery and Robotic Surgeyi and Educational, IEO (European Institute of Oncology)-IRCCS-Milan, Milan, Italy; 13 Department of Surgery, Rheinlandklinikum Lukaskrankenhaus Neuss, 84 41464 Neuss, Germany; 14 Oncologic surgery, Montpellier Cancer Institute, 34298 Montpellier, France

**Keywords:** Anastomosis, Colectomy, Complications, Endoscopy, Extracorporeal, Intracorporeal, Laparoscopy, Robot-assisted surgery

## Abstract

**Background:**

Right colectomy is the standard surgical treatment for tumors in the right colon and surgical complications are reduced with minimally-invasive laparoscopy compared with open surgery, with potential further benefits achieved with robotic assistance. The anastomotic technique used can also have an impact on patient outcomes. However, there are no large, prospective studies that have compared all techniques.

**Methods/design:**

MIRCAST is the Minimally-Invasive Right Colectomy Anastomosis Study that will compare laparoscopy with robot-assisted surgery, using either intracorporeal or extracorporeal anastomosis, in a large prospective, observational, multicenter, parallel, four-cohort study in patients with a benign or malignant, non-metastatic tumor of the right colon. Over 2 years of follow-up, the study will prospectively evaluate peri- and postoperative complications, postoperative recovery, hospital stay, and mid-term results including survival, local recurrence, metastases rate, and conversion rate. The primary composite endpoint will be the efficacy of the surgical method regarding surgical wound infections and postoperative complications (Clavien-Dindo grade III-IV complications at 30 days post-surgery). Secondary endpoints include long-term oncologic results, conversion rate, operative time, length of stay, and quality of life.

**Discussion:**

This will be the first large, international study to prospectively evaluate the use of minimally-invasive laparoscopy or robot-assisted surgery during right hemicolectomy and to control for the impact of the anastomotic technique. The research will contribute to current knowledge regarding the medical care of patients with malignant or benign tumors of the right colon, and enable physicians to determine which technique may be the most appropriate for their patients.

**Trial registration:**

This study was registered on Clinicaltrials.gov (clinicaltrials.gov identifier: NCT03650517) on August 28th 2018 (study protocol version CI18/02 revision A, 21 February 2018).

## Background

The standard surgical treatment for malignant neoplasms of the right colon is right colectomy (hemicolectomy). Open resection is associated with a relatively high rate of complications, but these may be reduced along with blood loss and hospital stay using laparoscopy [1, 2], a minimally-invasive technique in which operations are performed via small incisions (usually 0.5–1.5 cm) at a location distant to the site of interest. There are some limitations to the laparoscopic approach, including loss of a three-dimensional view, poor ergonomics, limited movement dexterity, and tremor [3], which may be overcome with robotic assistance. This minimally-invasive approach offers greater precision, flexibility, and control that minimizes the risk of injury to vessels and nerve structures, and enables oncologic resection [4]. Although longer operative times have been reported with robot right colectomy, the surgery can significantly reduce blood loss, postoperative complications and wound infections, lead to faster recovery of bowel function, fewer conversions to open surgery, and shorter hospital stay compared with laparoscopy [5, 6]. It should be noted that in many studies to date, the operating surgeon was often relatively inexperienced in robot-assisted surgery [5]; operative times can improve significantly with increasing experience [7]. There are also potential advantages in performing intracorporeal anastomosis (ICA, where the anastomosis is performed inside the abdominal cavity during minimally-invasive surgery) compared with extracorporeal anastomosis (ECA, where the anastomosis is performed by pulling out the bowel through a laparotomy). Benefits of ICA include smaller incision length, reduced time to first defecation, reduced short-term morbidity (e.g. less surgical wound infection), decreased rate of incisional hernias and re-interventions, and a shorter hospital stay compared with ECA [8, 9]. It is possible that the ability of the surgeon to perform ICA may be enhanced by robotic assistance, potentially reducing the conversion rate and improving the quality of suturing, but further research is required.

**Fig. 1:**
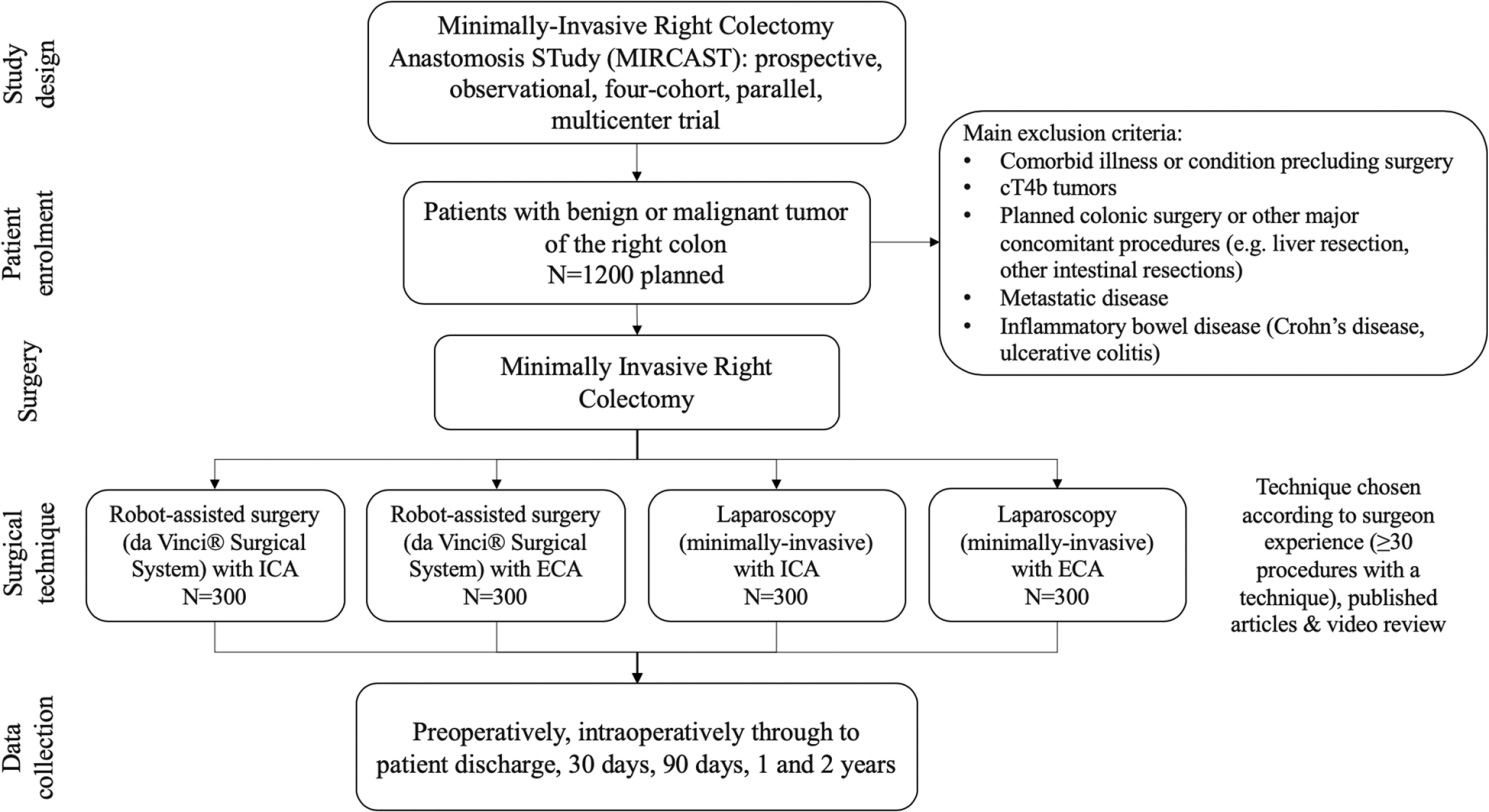
Study schematic. ECA, extracorporeal anastomosis; ICA, intracorporeal anastomosis

To date, many comparative studies of laparoscopic and robot-assisted right colectomy have been small, retrospective, non-randomized studies that did not control for anastomosis technique or the effect of robotic assistance on anastomosis. Prospective, multicenter studies that simultaneously compare both variables (i.e. surgical approach and anastomotic technique) are needed to provide evidence regarding the best technique for minimally-invasive right colectomy. Therefore, we have developed the Minimally-Invasive Right Colectomy Anastomosis Study (MIRCAST) (Fig. 1), a large prospective, observational, multicenter, parallel, four-cohort study designed to compare ICA and ECA after a minimally-invasive right colectomy, each using either laparoscopic and robotic-assisted approach, in patients with a benign or malignant, non-metastatic tumor of the right colon.

## Methods/design

### Patient recruitment

Patients included in the study will be adults (male or female aged ≥18 years) with a benign or malignant tumor in the right colon that require an elective right colectomy with curative intent. Patients will have a life expectancy of at least 12 weeks, with an adequate performance status (Eastern Cooperative Oncology Group Scale score ≤ 2). All patients will have voluntarily signed and dated an Ethics Committee-approved informed consent form before inclusion, obtained by the investigator or designee. Patients will be excluded if they have cT4b tumors, metastatic disease, planned colonic surgery along with other major concomitant procedures (e.g. liver resections, other intestinal resections), or have inflammatory bowel disease (i.e. Crohn’s disease or ulcerative colitis). Patients who are pregnant or suspected to be pregnant, have a comorbid illness or condition that would preclude the use of surgery, are undergoing emergency procedures, or are unwilling to comply with all follow-up study requirements will also be excluded. All patients will be screened and recorded during enrolment to identify any selection bias, which can be higher in observational studies [6].

Eligible patients will be recruited from the approved participating centers according to the surgeons’ experience with each technique. It is calculated that at least 1200 patients (300 per cohort) should be enrolled in the study. The assumptions for the sample size were based on the incidence of surgical wound infection and Clavien-Dindo grade > IIIa complications. The sample size is based on attaining a success rate of 85% for the primary endpoint, with the lower 95% confidence limit being no greater than 5% from the estimated success rate. Assuming a 2-sided interval, a total of 245–317 patients will be required within each cohort. Accounting for a 10% loss to follow-up, a total of 1200 patients will be recruited so that the four parallel cohorts will have sufficient power to make multiple comparisons.

### Study setting

This will be an international, multicenter study that will take place over a 4-year period, including 2 years of follow-up. The centers chosen will be high volume (recommended > 30 minimally-invasive right colectomies per year) and participating centers should ideally have an implemented enhanced recovery after surgery protocol. All investigators will be qualified colorectal or general surgeons experienced in the surgical management of patients with colon lesions and who have a patient population fitting the study requirements. Surgeons must select one cohort which is understood that is their standard approach. Surgeons will have had completed ≥30 minimally-invasive right colectomy procedures for a colon tumor using their chosen technique, have published their experience or contributed videos for review by the principal investigator and submit pictures of the surgical field when a D3 Lymphadenectomy is performed during the study, and will contribute and consecutively maintain their technique on all eligible subjects during the enrolment phase. Surgeons meeting mentioned criteria will be selected by their technique in order to balance the inclusion of 300 patients in each arm. Different surgeons from the same institution may select different techniques. Surgeons will be able to include patients in any cohort of the study for which they are qualified, and will be asked to enrol at least 15 cases per chosen cohort, per year. It is estimated that each surgeon will include 30 patients in one of the cohorts of the study. This means that we estimate to include 40 surgeons from different centres in Geographical Europe. The number of participating centres will also be close to 40 as in most of the centres only one experienced surgeon will participate in the study. It is desirable that participating centers have an implemented Enhanced Recovery After Surgery (ERAS) protocol although perioperative management including ERAS protocol is difficult to standardize in all multicenter studies.

A screening log with all right colectomies excluded from the study will be kept at all institutions and uploaded to the eCRF.

### Interventions

Patients will be recruited to one of four cohorts, depending on the surgeon’s experience: 1) Robotic right colectomy with ICA; 2) robotic right colectomy with ECA; 3) laparoscopic right colectomy with ICA; 4) laparoscopic right colectomy with ECA. For the ICA cohorts, Pfannenstiel incision will be the chosen wound for specimen extraction. If an operation cannot be completed using any of these minimally-invasive techniques, the patient will be considered a conversion to open surgery. Per protocol, conversion is defined as when anything apart of the anastomosis has to be done through the laparotomy. If the anastomosis is not completely performed in an intracorporeal approach during an Intracorporeal Anastomosis Case, the case is considered also converted.

MIRCAST Study is an observational study which has all the advantages and limitations of an observational study. One of the main limitations is that the surgical technique will not be standardized. Different factors (wound protectors, antibiotics, bowel prep…) might have an impact in the co-primary endpoint. As standardizing the technique is not an option in an observational study, all factors that might have an influence in the co-primary endpoint will be registered in the electronic Case Report Forms.

### Outcomes

The efficacy of the surgical method will be defined as the lack of surgical wound complications (infection, hematoma, hernia as late complication of wound infection…) and reduced severe complications. The primary composite endpoint will comprise two measures of success: Having either a surgical wound infection or a Clavien IIIb-IV postoperative complication constitute the primary composite endpoint. This means, that a patient having either a surgical wound infection or a Clavien IIIb-IV [10] complication would be scored as a “1”. Surgical wound infection will be assessed at the 30 postoperative day visit using specific wound assessment questions (purulent drainage, positive wound cultures, wound opened to drain and classical flogotic signs). These questions are recorded at the eCRF (Yes recorded as 1, No recorded as 0). In all cohorts, secondary endpoints will include oncologic results at 2 years, overall survival, disease-free survival, local recurrence, metastases rate, rate of unplanned conversions to open surgery, operative time (min), complete mesocolic excision, number of harvested lymph nodes, R0 resection, length of stay (days), ventral hernia (assessed 1 and 2 years after the right colectomy), patient-assessed quality of life measures (the European Organization for Research and Treatment of Cancer Quality of Life Questionnaires C30 and CR29), and systemic inflammatory response syndrome (C-reactive protein, with optional procalcitonin). Optional measures, to enable a medico-economic sub-study, include EQ-5D, time to ambulation, patient return to work/activity, pain evaluation, and resource utilization for procedure and follow-up care.

### Complications

All study techniques are in practice today and only surgeons competent in a procedure will be allowed to perform those surgeries. Therefore, there are no anticipated additional risks than would normally be encountered in these patients. All complications will be captured on the electronic complication case report form, including in detail signs and symptoms related with wound infection (tenderness at the wound, swelling around the wound, redness of the wound, heat of the wound). All complications will be graded by the investigator according to the Clavien-Dindo classification [10]. The investigator or designee will monitor the occurrence of complications associated with the surgical procedure, as well as procedure-related and postoperative clinical sequelae; the principal investigator will report complications to the local ethics committee and, if related to the robotic system, to the Initiative EMEIA Complaints Team and to the study sponsor.

### Data collection and management

**Fig. 2:**
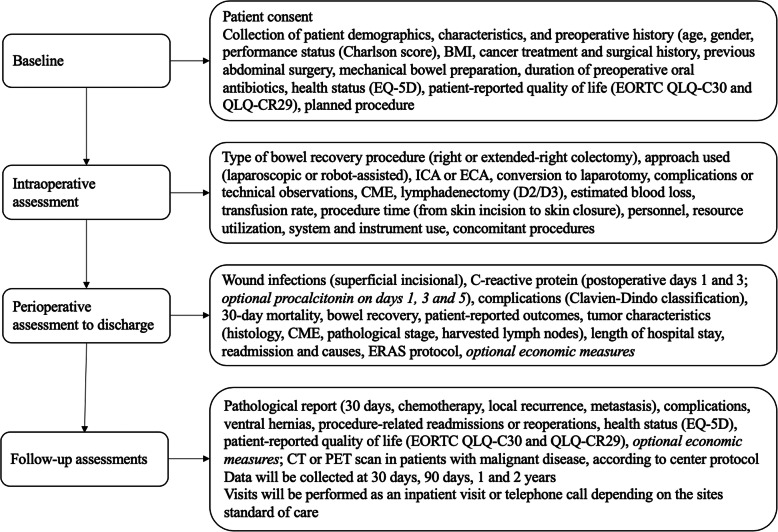
Study assessments. BMI, body mass index; CME, complete mesocolic excision; CT, computed tomography; ECA, extracorporeal anastomosis; ERAS, enhanced recovery after surgery; EORTC, European Organization for Research and Treatment of Cancer; ICA, intracorporeal anastomosis; PET, positron emission tomography; QLQ-C, quality of life questionnaire for cancer patients; QLQ-CR, quality of life questionnaire for colorectal cancer patients

Data will be collected prospectively according to the schedules outlined in Fig. 2 and Table 1. Enrolled patients will undergo assessments at the following intervals: preoperatively, intraoperatively through to discharge, 30 days, 90 days, 1 and 2 years post-procedure. At baseline and once patient consent has been obtained, patient demographics and medical history (including indication and planned procedure) will be collected. Patient-related quality of life and functional measures will be completed by the patient. Data related to the operative procedure itself will be collected, including procedure timing, personnel, resource utilization, system and instrument use, need for conversion, complications and technical observations. During the perioperative period until discharge, data collected will include wound infection, complications, 30-day mortality, length of stay, and tumor characteristics. During follow-up, patient-related functional outcomes and quality of life measures will be completed and any complications or protocol deviations reported, according the standard follow-up procedure for each center.

**Table 1:** Assessment schedule

	Baseline	Procedure	Discharge	30 days ±7 days	90 days ±14 days	1 year ±30 days	2 years ±30 days	Unscheduled visit
Informed consent	X							
Demographics and medical history	X							
Procedure details		X						
SIRS (CRP) *Optional: PCT*		X						
Pathology				X				
CT scan/PET scan^c^	X					X	X	
Complications		X^a^	X^a^	X^a^	X^a^	X^a^	X^a^	X^a^
Quality of life: EQOL	X				X	X		
Study exit forms							X^b^	

^a^Complete if applicable; ^b^complete when lost to follow-up, consent withdrawal or subject has completed all study related visits; ^c^CT scan results will be collected if done per the sites standard protocol: every 6 months for malignant disease; after at least 1 year to assess hernia in benign disease. *CRP* C-reactive protein (collected at days 1 and 3 postoperative); *CT* computerized tomography, *PCT* procalcitonin (optional, collected at days 1, 3, 5 postoperative); *PET* positron emission tomography, *SIRS* systemic inflammatory response syndrome

Primary data collection will be performed by a study coordinator or designee. The data will be collected on the electronic case report forms (CRF), hosted in a study-specific, dedicated electronic data collection (EDC) system. The database has been developed and utilized in accordance with international requirements and standards applicable to clinical investigations i.e. good clinical practice (GCP) and is a GCP-compliant environment meeting applicable 21 CFR Part 11 requirements. A copy of the completed CRF will remain on site at the participating center at the end of the study. All data requested on the CRFs will be recorded. All missing data will be explained. The European Society of Coloproctology will conduct two independent snapshot audits during the study, during the first and second years, which will also collect information on all right colectomies performed in the participating institutions.

Study monitoring will be conducted using a risk-based monitoring approach (i.e. most monitoring activities will be performed using remote monitoring functionality by reviewing the data from the EDC system). The on-site monitoring will be conducted on an as-needed basis in situations including, but not limited to, protocol compliance issues, major data discrepancies, and safety issues. In general, the study monitoring functions will be performed by the sponsor or its appointed designee in compliance with recognized GCP, the harmonized standard EN ISO 14155, and local applicable legislation. The major function of the clinical monitor is to observe and assess the quality of the clinical study. It will be the responsibility of the site-appointed research personnel to complete all CRF and to document conformity to the clinical trial protocol throughout the study.

There will be no study-specific tests, either preoperatively or postoperatively, that are beyond what is routine and customary for patients undergoing the surgical procedures under consideration. Patients can choose to withdraw at a time, and the investigator has the right to discontinue patients at their discretion to ensure their wellbeing. In the event of a patient’s early withdrawal from the study, all CRF will be obtained up to the point of withdrawal, with the reason for withdrawal documented, and no further follow-ups will be performed. If a patient cannot be reached during a visit window, a missed visit will be recorded; after three consecutive missed visits, a patient will be considered lost to follow-up and a study exit form will be completed in the electronic CRFs. All protocol deviations will be recorded on the protocol deviation form.

### Statistical analysis

All analyses is detailed in a statistical analysis plan. Patients from each cohort will be analyzed separately on an intent-to-treat basis. Descriptive statistics will be provided for all discrete variables in the form of rates and proportions with 95% confidence intervals (CI). Continuous variables will be escribed by mean, standard deviation, median and range. Overall survival, disease-free survival, local recurrence rate and metastasis rate will be estimated using the method of Kaplan Meier. Exploratory comparisons of discrete variables will be performed using a Chi-squared test, using continuity correction or Fisher’s exact test. Continuous variables will be compared using a Student’s t-test, or a non-parametric equivalent (Wilcoxon). Survival statistics will be based on a stratified log-rank test. All tests will be two sided with a *p*-value of less than 0.05 considered to indicate statistical significance.

An interim analysis is planned after having recruited 300 patients, 75 patients at least per cohort, in order to evaluate whether a success rate around 85% is achieved for each cohort; a lower limit of 75% is not acceptable, for which reason the data from the treatment groups with a success rate < 75% will be excluded from further analysis. None a priori expectations of differences can be made based in current literature.

A propensity score will be constructed as the individual prediction (in logit scale) of a logistic regression model in which the outcome is modeled with different potential confounding factors. The propensity score will be added as a continuous variable to adjust the risk models. The Generalized Linear Model (GLM) approach allows for the construction of multilevel and mixed models, which will be useful for controlling the inter-hospital random variability.

Secondary end-points will be analysed according to their characteristics: GLM with identity link will be used for operative time, number of harvested lymph nodes and length of stay, GLM with logit link will be used for unplanned conversion, complete mesocolic excision and R0 resection and Cox regression will be used for middle-term oncologic results: overall survival, disease-free survival, local recurrence and metastasis.

Patients with missing data in planned surgical approach will be be excluded from the study. Patients with missing data in any end-point will be excluded from the analyses on that end-point, and patients with missing data in variables included in the propensity score will have their data imputed using multiple imputation procedures.

Three sensitivity analyses will be used for the primary end-points. The analysis will be repeated k times omitting a hospital each time (k = number of participant hospitals). In addition to intention-to-treat analysis, a sensitivity analysis will be conducted excluding patients with conversion. The analysis will be repeated stratifying by tertiles of the propensity score.

Stata/SE software will be used to perform the statistical analysis (versions 14–16 [forthcoming]).

### Sample size calculation

The assumptions for the sample size of this study have been developed based on the incidence of Surgical Wound Infection, as well as the incidence of Clavien-Dindo grade > III complications as seen in the Introduction. The sample size is based on attaining a success rate of 85% for the primary endpoint, with the lower 95% confidence limit being no greater than 5% from the estimated success rate. Assuming a 2-sided interval, a total of 245–317 subjects will be required within each cohort. Accounting for a 10% loss to follow up, a total of 1200 subjects will be recruited for this study. Several interim analysis are planned through the study in order to build new power analysis and adjust sample size accordingly if needed.

The design of this study envisages four parallel cohorts for which the sample size has been developed for each cohort according to a precision around an expected success rate with enough subject and sufficient power to make multiple comparisons within the appropriate method. Any statistical inferences drawn based on pairwise comparisons will be undertaken in a posteriori statistical analysis using a propensity score to account for confounding covariates.

The primary endpoint for success is defined as a patient meeting the following criteria:

- Surgical Wound Infection 5% lower in the ICA group.
- Grade IIIb-IV Clavien -Dindo complications at 30 days post op 5% lower in the ICA group.

According to the current literature the the following numbers represent Surgical Wound Infection and Severe Complications in intermediate volume centers:

**Table:** 

ICA		ECA
Surgical Wound Infection	_4–5%_^5,8,10^	_10–14%_^5,8,10^
Morbidity Dindo 3b-4	1.1–5%^5,8^	8–11%^5,8^

### Ethics and dissemination

The study will be conducted in accordance with applicable European Regulatory requirements, the Declaration of Helsinki, and the principles of GCP Guidelines. Ethics Committee approval of the study protocol, patient informed consent form, and other required study documentation, with all other country-specific approvals, will be obtained for the study prior to study initiation at the site. Initial Ethical Committee approval will be obtained at Institutional Review Board (Ethics Committee) Of Cantabria (CEIC).

Information about the study patients will be kept confidential and managed according to the requirements of the Health Insurance Portability and Accountability Act of 1996 and the European Standards on Confidentiality and Privacy in Healthcare. Patient confidentiality will be strictly maintained. Patients will be assigned a study identification. Access to patient records will be limited to the study investigator, the investigator-delegated study coordinator, and clinical representatives from Intuitive Surgical. Provision for review of the data by governmental agencies and Intuitive Surgical will be incorporated into ethics committee submissions.

### Study websites

www.mircast.net

https://www.escp.eu.com/research/cohort-studies/2019-escp-supported-robotics-projects-mircast-and-reset

## Discussion

Laparoscopy has been shown to have advantages over open resection in the surgical treatment of neoplasms of the right colon [1, 2], but additional benefits may be achieved using robotic assistance [5, 6]. However, most studies to date have been small and retrospective, with no control for the anastomosis technique used. Large, adequately-powered, prospective, multicenter studies are required to provide evidence regarding the best approach to use. MIRCAST was designed to address these issues, and will be the first to not only compare laparoscopy against robotic-assisted surgery, but to also assess the impact of using ICA versus ECA. There is published data to suggest that ICA is associated with reduced post operative ileus and less pain as compared to ECA and also allows the surgeon to choose an extraction site in the lower abdomen. Still the uptake of this technique amongst colorectal surgeons’ has been limited primarily due to the technical difficulties of doing this laparoscopically and the worry for risk for peritoneal contamination and infection. However a robotic platform can make this a much easier task with the ability to perform a stapled anastomosis and suture close the enterotomy in small bowel and colon. Although a randomized controlled trial would be ideal, it is not feasible to randomize patients as not all centers in Europe have access to robotic systems or have training in ICA. Each surgeon will be experienced in their chosen procedure, to ensure that all patients within each cohort receive optimal treatment and participating centers will have ERAS protocols implemented for the perioperative care of the patients. The study will focus on perioperative complications (including surgical wound infections and postoperative complications, in a composite primary endpoint that will offer greater statistical precision and efficiency [11], as well as potential benefits of robotic-assisted surgery for right colon resections. For example, it has been suggested that use of a robotic platform may be advantageous in right hemicolectomy, which requires a wide range of colon resections and involves a more complex vascular anatomy than left colectomy [6]. A more aggressive surgical approach is also required in cancer patients, in whom it is necessary to follow the principles of oncologic radicality (i.e. complete mesocolic excision). Finer and more gentle tissue manipulation and dissection is possible with robotic assistance, which allows the operative field to be magnified and offers precise instrument control [6]. This may, for example, translate to the lower intraoperative blood loss that has been observed with robotic surgery compared with laparoscopy [5, 6]. Robotic assistance may also help a surgeon to perform ICA. Practical aspects of each minimally-invasive technique will also be evaluated, including operative time (often reported to be longer with robot-assisted surgery), length of hospital stay and unplanned conversion to open surgery. The impact of surgery on patients’ quality of life will also be determined. The research performed in the MIRCAST study is expected to contribute to the clinical data on the medical care of benign or malignant tumors of the right colon using minimally-invasive surgery.

## Data Availability

Protocol available at ClinicalTrials.gov (ClinicalTrials.gov Identifier: NCT03650517). The datasets generated and/or analysed during the current study are not publicly available due Data Protection and Access agreements signed with the different participating Institutions but will be available from the corresponding author on reasonable request after outcomes of the study are published.

## Abbreviations

MIRCAST: Minimally-Invasive Right Colectomy Anastomosis Study
ICA: Intracorporeal Anastomosis
ECA: Extracorporeal Anastomosis
ERAS: Enhanced Recovery After Surgery
eCRF: Electronic Case Report Forms
EDC: Electronic Data Collection
EQ-5D: EuroQuality of Life 5 Dimensions
CR: Colorectal
EMEIA: Europe, Middle East, India and Africaç
GCP: Good Clinical Practice
CI: Confidence Interval
GLM: Generalized Linear Model
EC: Ethics Committee
